# Advancements in Polymer Biomaterials as Scaffolds for Corneal Endothelium Tissue Engineering

**DOI:** 10.3390/polym16202882

**Published:** 2024-10-12

**Authors:** Kevin Y. Wu, Myriam Belaiche, Ying Wen, Mazen Y. Choulakian, Simon D. Tran

**Affiliations:** 1 Department of Surgery, Division of Ophthalmology, University of Sherbrooke, Sherbrooke, QC J1G 2E8, Canada; yang.wu@usherbrooke.ca (K.Y.W.);; 2 Faculty of Medicine, University of Montreal, Montreal, QC H3T 1J4, Canada; 3 Faculty of Dental Medicine and Oral Health Sciences, McGill University, Montreal, QC H3A 1G1, Canada

**Keywords:** corneal tissue engineering, polymer scaffolds, human corneal endothelial cells (CECs), biocompatible biomaterials, corneal endothelial regeneration, 3D bioprinting in ophthalmology, synthetic polymers for corneal repair, micro- and nano-topologies, drug delivery systems for corneal endothelium

## Abstract

Corneal endothelial dysfunction is a leading cause of vision loss globally, frequently requiring corneal transplantation. However, the limited availability of donor tissues, particularly in developing countries, has spurred on the exploration of tissue engineering strategies, with a focus on polymer biomaterials as scaffolds for corneal endotlhelium regeneration. This review provides a comprehensive overview of the advancements in polymer biomaterials, focusing on their role in supporting the growth, differentiation, and functional maintenance of human corneal endothelial cells (CECs). Key properties of scaffold materials, including optical clarity, biocompatibility, biodegradability, mechanical stability, permeability, and surface wettability, are discussed in detail. The review also explores the latest innovations in micro- and nano-topological morphologies, fabrication techniques such as electrospinning and 3D/4D bioprinting, and the integration of drug delivery systems into scaffolds. Despite significant progress, challenges remain in translating these technologies to clinical applications. Future directions for research are highlighted, including the need for improved biomaterial combinations, a deeper understanding of CEC biology, and the development of scalable manufacturing processes. This review aims to serve as a resource for researchers and clinician–scientists seeking to advance the field of corneal endothelium tissue engineering.

## 1. Introduction

The corneal endothelium is vital for maintaining corneal transparency and hydration, essential for clear vision. Diseases related to corneal endothelial dysfunction, such as Fuchs’ dystrophy or pseudophakic bullous keratopathy, result from a reduction in endothelial cell density, eventually resulting in corneal edema and compromised vision [1]. These conditions often necessitate corneal transplantation, but the global shortage of donor corneas, particularly in developing regions, underscores the need for alternative treatment strategies [2]. Corneal transplantation remains the standard treatment for severe irreversible corneal diseases; however, fewer than 2% of patients requiring a transplant are able to receive one due to the significant shortage of donor corneas. With a staggering 70:1 disparity between available donors and patients, particularly in regions such as India, China, and parts of Africa, this shortage is expected to worsen unless innovative treatments are rapidly developed [2].

Tissue engineering has emerged as a promising solution, with polymer biomaterials at the forefront of developing scaffolds for corneal endothelium regeneration. These scaffolds must replicate the native Descemet membrane’s properties to support corneal endothelial cell (CEC) growth and functionality [3,4].

This review provides an overview of recent advancements in polymer biomaterials for corneal endothelium tissue engineering. Key topics include the essential scaffold properties—including but not limited to optical clarity, biocompatibility, mechanical stability, and permeability—and an evaluation of natural, synthetic, and semi-synthetic polymers. We also explore innovations in micro- and nano-topologies, fabrication techniques like electrospinning and 3D/4D bioprinting, and the integration of drug delivery systems.

While progress is evident, challenges remain in translating these technologies to clinical practice. This review aims to offer insights into overcoming these hurdles, guiding future research toward effective and accessible treatments for corneal endothelial dysfunction.

Table of contents: The table outlines key areas of focus, including the structure and disorders of the corneal endothelium, corneal transplantation techniques, essential properties of biomaterials, and the application of polymer biomaterials in corneal tissue engineering. Emerging technologies and future challenges are also highlighted. **Section****Subsections**The Corneal Endothelium- Structure and Function - Primary Endothelial Disorders - Secondary Endothelial DisordersCorneal Transplantation Techniques- Penetrating Keratoplasty (PK) - Endothelial Keratoplasty (EK) - DSAEK and DMEK - Bioengineered CorneasEssential Properties of Polymer Biomaterials for Corneal Endothelial Implants- Descemet’s Membrane (DM) Characteristics - Key Scaffold PropertiesPolymer Biomaterials for Corneal Endothelium Tissue Engineering- Natural Tissue Substrates - Natural and Semi-Synthetic Polymers - Synthetic PolymersMicro- and Nano-Topological Morphologies- Importance of Surface Morphology - Techniques for Creating StructuresEmerging Technologies and Innovation- Peptide and Electroconductive Hydrogels - 3D and 4D Bioprinting - Scaffolds with Drug Delivery SystemsChallenges and Future Directions- Current Limitations - Areas for Further Research - Need for Clinical Translation

## 2. The Corneal Endothelium 

**Figure 1:**
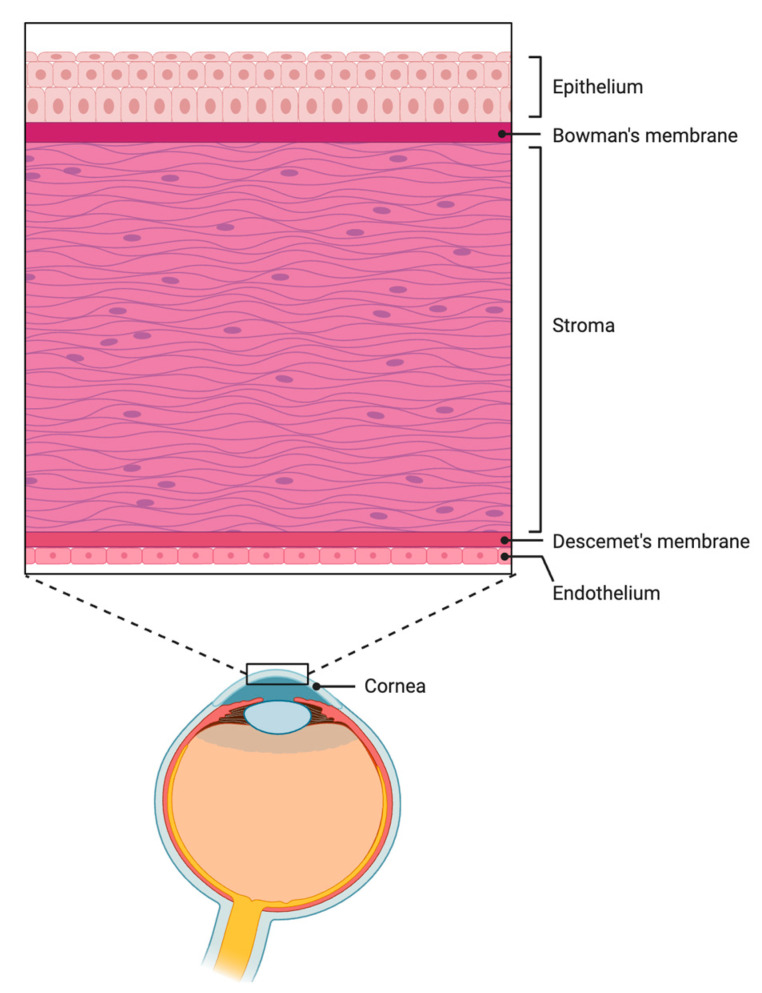
Histology of the human cornea. Created with BioRender.com.

The corneal endothelium consists of a monolayer of interconnected hexagonal cells that line the posterior surface of the cornea. Its main functions are to maintain the proper hydration level and nutrition of the stroma in order to ensure its structure and clarity for normal vision. The endothelium is permeable to nutrients diffusing passively from the aqueous humor to the stroma, while actively balancing the diffusion gradient with its ion transport system known as the endothelium pump [5]. Human corneal endothelial cells do not have the capacity to regenerate, decreasing throughout life from a density of approximately 5000 cells/mm^2^ at birth to 2400 cells/mm^2^ in adulthood [6]. The basement membrane of the corneal endothelium, Descemet’s membrane, is a collagen layer secreted by corneal endothelial cells. Continuous production by the endothelium causes DM to thicken throughout life, reaching 10–12 um in adulthood [6]. Figure 1 illustrates the different layers of the human cornea.

Damage to corneal endothelial cells leads to decreased pump function and increased permeability of the corneal endothelium. Edema of the stroma occurs as a consequence, causing the loss of transparency of the cornea. There exist several primary disorders of the corneal endothelium, with the most common being Fuchs’ endothelial corneal dystrophy (FECD). It is the leading cause of corneal transplantation around the world [7]. FECD is a progressive bilateral disease characterized by the formation of corneal guttae followed by stromal edema and a subsequent decrease in vision [5,6,8].

Other primary disorders include posterior polymorphous dystrophy (PPMD), congenital hereditary endothelial dystrophy (CHED), and iridocorneal endothelial syndrome (ICE). PPMD and CHED are both rare bilateral hereditary disorders. The former is characterized by progressive corneal edema and opacification with epithelial-like endothelial cells, while in the latter, diseased corneal endothelium is present at birth [6,9,10]. ICE group disorders are characterized by the proliferation of endothelial cells toward the iris, leading to corneal edema and corectopia [11].

Corneal endothelial cell loss can also be secondary to other pathologies. The intraocular pressure increase in glaucoma can lead to endothelial cell loss [12]. Diabetes mellitus has also been found to damage corneal endothelial cells. It has been associated with increased changes in cell morphology and cell death, leading to corneal edema [13]. Pseudophakic bullous keratopathy is another well-known secondary corneal disorder. It is characterized by trauma to endothelial cells, leading to irreversible corneal edema following intraocular surgery [6,14].

**Figure 2:**
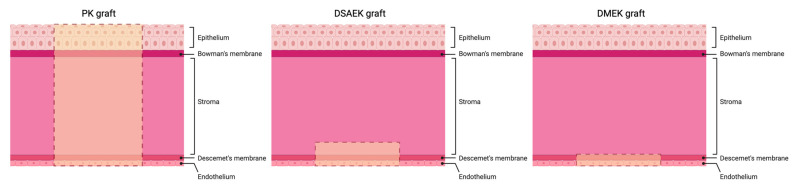
Penetrating keratoplasty (PK), Descemet’s stripping automated keratoplasty (DSAEK), and Descemet’s membrane endothelial keratoplasty (DMEK). Created with BioRender.com.

Corneal transplantation is currently the standard treatment for corneal endothelium disease. Penetrating keratoplasty (PK), a full-thickness replacement of the cornea, was first developed in 1905 and has been widely performed [15]. However, PK carries a certain risk of complications, such as expulsive hemorrhage and increased incidence of graft rejection, due to its invasive nature. Additionally, patients undergoing PK often face a longer recovery period, with potential fluctuations in post-operative visual acuity. Endothelial keratoplasty (EK) is a newer, more selective method of corneal transplantation that has taken over PK for endothelial pathologies. In Descemet’s stripping automated keratoplasty (DSAEK), the diseased endothelium and DM are dissected with a microkeratome and replaced by donor stroma, DM, and endothelium. In the more recent Descemet’s membrane endothelial keratoplasty (DMEK), the host’s DM and endothelium are replaced by a donor DM and endothelium only. DMEK’s thinner graft allows for a faster recovery time and has a decreased graft rejection rate, but also has a higher risk of graft detachment [16,17]. Since 2022, DMEK has become the most commonly used technique, surpassing DSAEK due to its superior outcomes [18]. The different techniques can be visualized below in Figure 2. 

### Bioengineered Corneas: An Urgent Solution to the Donor Shortage

Corneal transplantation remains the standard treatment for severe irreversible corneal diseases. However, a global shortage of donor corneas presents a significant obstacle, with fewer than 2% of patients requiring a transplant able to receive one. The disparity between available donors and patients on the waitlist is a staggering 70:1 worldwide, particularly in rapidly growing populations such as those in India, China, and parts of Africa. This shortage is expected to worsen unless innovative treatments for corneal blindness beyond transplantation are rapidly developed [2]. In response, bioengineered corneal tissue has emerged as a promising alternative, representing a critical focus for future research and development.

**Figure 3:**
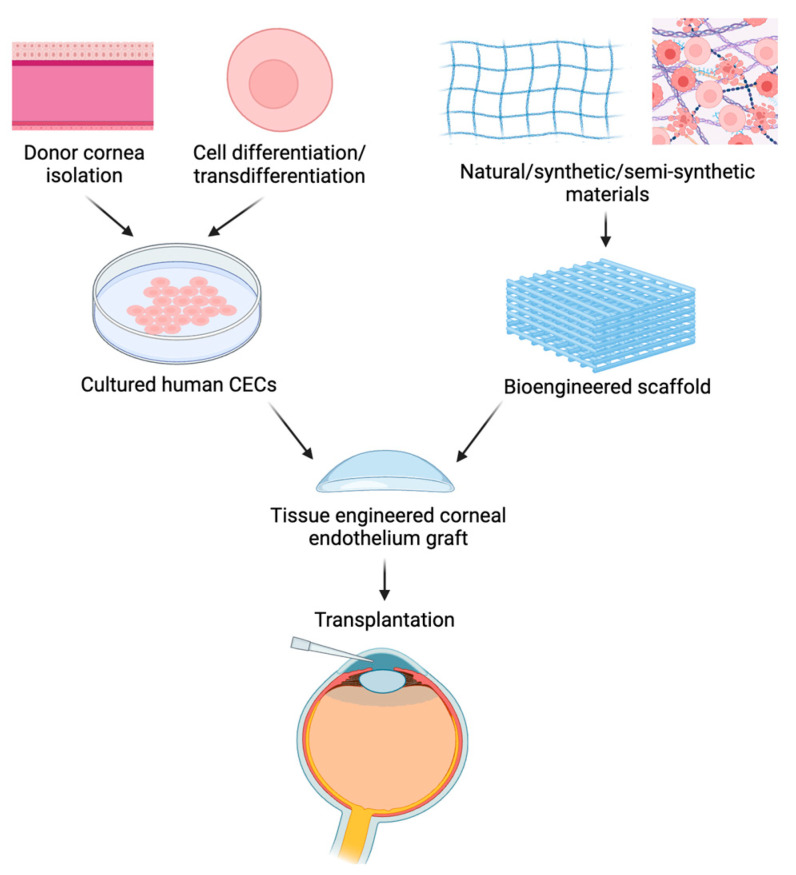
Corneal endothelium tissue engineering. Created with BioRender.com.

With increasing demands for corneal transplantation and a lack of cornea donors worldwide, there is a crucial need to find alternative solutions to donor tissues. Advances in regenerative medicine are important to relieve the shortage of donors. In the last two decades, studies have focused on corneal endothelial cell (CEC) cultures and their expansion in vitro from sources such as human donor cornea isolation, pluripotent stem cell differentiation, and cell transdifferentiation. The cultured CECs can then be delivered into the host eye by cell injection or tissue-engineered grafts. The emerging field of corneal endothelium tissue engineering constructs grafts by assembling cultured CECs onto a scaffold for transplantation (see Figure 3). Scaffolds play a crucial role in ensuring the viability of the graft, and many materials, whether derived from natural tissue or developed from polymers, are being studied to meet the characteristics needed for a functional scaffold [16,19,20,21].

## 3. Essential Properties of Polymer Biomaterials for Corneal Endothelial Implants

**Figure 4:**
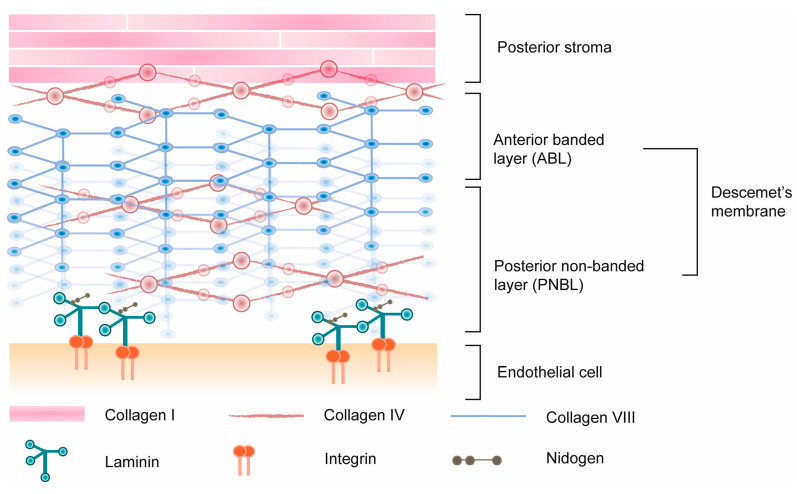
Schematic of the ECM structure of Descemet’s membrane [25]. Reprinted with permission from ref. [25]. Copyright 2021 Elsevier.

DM is a dense layer secreted by the corneal endothelium and serves as its basement membrane [22]. Its extracellular matrix (ECM) is a network primarily composed of collagen IV, laminin, fibronectin, collagen VIII, nidogens, and perlecan [3,23,24]. Integrins enable the interaction between corneal endothelial cells and those components, as illustrated in Figure 4 [25]. DM provides structural support and biochemical cues essential for CEC behavior, such as adhesion, migration, and proliferation, and it ensures that corneal transparency and function is preserved [3,4]. It is imperative that scaffolds for CECs mimic the biomechanical properties of the native DM to promote proper cell integration and functionality. Those properties include biocompatibility, biodegradability, mechanical stiffness, transparency, permeability, and other essential characteristics.

### 3.1. Optical Clarity and Transparency

Transparency is the ability to transmit light. It can be effectively examined ex vivo by spectrophotometry [26]. The scaffold must have a transparency of at least 90%, which is the transparency of the corneal DM [27], and a refractive index as close to the corneal stroma as possible (approximately 1.375) for optimum visual acuity. A slight change in those parameters can lead to scattering and poor clarity [28]. Furthermore, a healthy cell density ranging between 2600 and 4000 cells/mm² is also important for the proper control of fluids and thus transparency, since it facilitates the creation of tight junctions and the adequate functioning of Na+/K+-ATPase pumps [29]. It is important to note that achieving the required optical clarity remains challenging for human amniotic membranes [27], silk fibroin (when used in its native form without any modifications) [30], and materials produced by electrospinning [31].

### 3.2. Biocompatibility and Biodegradibility

Most natural materials, such as gelatin and chitosan, are biodegradable. Polycaprolactone, the most commonly used synthetic material with a relatively slow degradation rate, is also biodegradable. Non-biodegradable materials such as polymethylmethacrylate can also be employed if the implant is meant to be permanently present in the body [32,33] , but they are more rarely used due to long-term safety and inflammatory responses. If utilized, they must undergo cytotoxicity tests and it must be ensured that they break down at the same rate that the ECM regenerates [29]. An efficient way to measure biodegradation in vitro is to use collagenase digestion assays, as seen in various studies [27,34]. A few examples of promising biodegradable grafts include the combination of poly(lactide-*co*-caprolactone) (PLCL) to an extracellular matrix (ECM) derived from in vitro cultured mesenchymal stem cells found in umbilical cord blood [35] and biomedical elastomers [36]. 

### 3.3. Mechanical Properties and Stability 

Scaffolds’ mechanical stiffness is another crucial property in tissue engineering [37]. Different tools are used to assess mechanical stability, such as tensile strength, Young’s modulus, elongation properties, and thickness. In addition to ensuring the implant can withstand deformation and retain its properties, whether during implantation or once unfolded into place, it also significantly influences CEC behavior and morphology. Recent research demonstrates that softer substrates generally display more preserved pluripotency markers (like Oct4 and Nanog) and are better for maintaining undifferentiated states by inhibiting the Paxillin-YAP signaling pathway, while stiffer substrates promote the differentiation and endothelial–mesenchymal transition (EnMT) that are necessary to ensure effective corneal restoration [38]. Additionally, a study by Guo et al. (2006) showed that cells are more likely to adhere and migrate on stiffer substrates, which is essential for the initial attachment and covering of the scaffold by CECs [39]. However, matrix stiffness does not only influence the properties of individual cells, but also the communication between cells, with slightly softer substrates having more frequent cell–cell interactions [40]. This suggests that incorporating slightly softer regions or gradients within the substrate may optimize the coordination between cells and tissue integrity. The implant should also have a sufficient level of viscoelasticity to enable its adaptation to the changing curvature of the cornea during healing. Ultimately, as for the other properties, scaffolds’ mechanical properties should closely match those of DM. An experimental study by Danielsen (2004) showed that human DM has a tensile strength of approximately 1.72 MPa, a Young’s modulus of 11.8 MPa, and a maximal strain of 0.312 [41]. As for DM’s thickness, it ranges between 10 and 12 μm [6]. Du et al. (2022) showed that when exposed to these physiological conditions, CECs display better cell density and viability, a more stable cytoskeleton, and stronger expression of functional markers than softer or stiffer materials [42].

### 3.4. Permeability and Nutrient Transport 

Permeability is another crucial property in corneal endothelium tissue engineering. It refers to the scaffold’s ability to transport liquids, gasses, and nutrients, which ensures the metabolic needs are met. However, a balance needs to be established between this parameter and the preservation of corneal dehydration, since excessive liquid buildup leads to swelling and a lack of transparency. There is very little information available on the diffusion coefficient of DM. Nonetheless, permeability can be assessed with corneal diffusion coefficients. The corneal diffusion coefficient of glucose is approximately 3.0 ± 0.2 × 10^–6^ cm^2^/s [43]. Furthermore, Charalel et al. (2012) established that the diffusion coefficient for bovine serum albumin through the human cornea is 3.1 × 10^−^8 cm^2^/s, with a 95% confidence interval ranging from 2.1 × 10^−8^ to 4.1 × 10^−8 ^cm^2^/s [44]. In addition, the use of quantitative structure–permeability relationships (QSPR) is gaining momentum. These models can help design scaffolds with optimal permeability by relating physicochemical properties and tissue thickness to nutrient and drug transport. This tool can also improve the bioavailability of various treatments, as well as drug delivery systems within scaffolds, an emerging innovation which will be further discussed in Section 6 [45].

### 3.5. Surface Wettability and Hydrophilic/Hydrophobic Nature 

Hydrophobicity is a crucial aspect of biomaterials that can optimize a scaffold for various applications. Ayala et al. (2011) studied hydrogels with different alkyl chain lengths (C1 to C10) showing varying hydrophobicities. Their study showed that matrices with 4–6 CH2 groups, particularly C5 hydrogels, enabled optimal adhesion, cell motility, and the cellular organization of human mesenchymal stem cells (hMSCs) while supporting both myogenic and osteogenic differentiation even without external osteogenic-inducing soluble factors. This highlights the significant impact of matrix hydrophobicity on cell behavior and on scaffold design in regenerative medicine [46]. A wealth of other studies show that hydrophobicity affects cell adhesion and proliferation [47,48]. Furthermore, as shown by Larrañeta et al. (2018) and McKenzie et al. (2015), modulating this property in typically hydrophilic hydrogels could allow them to deliver hydrophobic drugs, which could improve targeted therapies [49,50]. However, while moderate hydrophobicity can enhance mechanical stability and support long-term cell adhesion, proliferation, and phenotype preservation, superhydrophobic surfaces were found to inhibit cell proliferation and protein absorption [51]. As for hydrophilic materials, they are generally biocompatible and can promote cell adhesion and nutrition transport. However, extreme hydrophilicity can lead to poor cell attachment and proliferation, as seen in the effect of polyvinyl alcohol on bovine corneal endothelial cells in a study realized by Wang et al. (2011) [52]. This highlights the need to balance hydrophilicity and hydrophobicity to obtain the desired CEC behavior.

### 3.6. Ability of the Scaffold to Maintain the Differentiated State of CECs

In addition to mechanical and biophysical properties, CECs must maintain their differentiated states in vivo to ensure successful transplantation. Tools to characterize CECs include the preservation of their hexagonal morphology and the expression of phenotypic markers, the most common ones being Na^+^/K^+^ ATPase, zonula occludens-1 (ZO-1), Ki67, and collagen type VIII. However, these indicators are not specific to CECs and there is a lack of robust identification techniques. While CD166, CD200, GPC4, HLA-ABC, and PD-L1 are phenotypic markers that have been associated with healthy CECs [53,54,55,56], there is still a lack of consensus on their specificity. Those are important obstacles to the advancement of research on cultured corneal endothelial cells to in vivo or clinical trials, as will be discussed in Section 7 [57]. Moreover, a study by Parekh et al. (2019) showed that with the assistance of Viscoat, passaged CECs have higher proliferative and adhesive capacities compared to CECs that are left to attach without the viscoelastic solution. They also display better confluence rates and higher preservation of their hexagonal morphology and differentiated state markers, such as CD166, PRDX-6, Na^+^/K^+^-ATPase, ZO-1, and Ki-67 [58]. Another study by Frausto et al. (2020) showed that low-mitogenic culture conditions are better than high-mitogenic conditions in helping CECs preserve their cell circularity, as well as their phenotypic and functional characteristics. They also help CECs have a more robust functional barrier and prevent senescence-associated morphogenesis [59]. Designing scaffolds that replicate these culture conditions has the potential to optimize the maintenance of CEC health and functionality after transplantation.

Surface properties are also vital components in the design of corneal endothelium scaffolds, and this will be discussed in detail in Section 5.

## 4. Polymer Biomaterials for Corneal Endothelium Tissue Engineering

### 4.1. Natural Tissue Substrates

Scaffold substrates can be derived from the extracellular matrix of natural tissue membranes. Previously studied sources include human amniotic membranes, decellularized lens capsules [27], decellularized corneal stroma [60], porcine Descemet’s membrane [61], and fish scales [62]. A recent study by Zhao et al. (2022) attempted to enhance the mechanical strength of amniotic membrane scaffolds through corneal crosslinking and modification with Descemet’s membrane components. The endothelium grafts were transplanted in vivo by DMEK in cat and monkey subjects, and they were able to maintain normal structure and transparency, showing potential for future clinical application [63].

### 4.2. Polymer Substrates

Polymer biomaterials are frequently used in tissue engineering, and they can come from natural or synthetic sources. Natural polymers have great biocompatibility, cell adherence, and cell differentiation properties. However, they can be inconsistent due to their biologic variability and contribute to varying scaffold properties. Synthetic polymers, on the other hand, are very easy to customize and reproduce in big quantities, ensuring consistent results. They also have the necessary mechanical and chemical properties needed for a proper scaffold, but they are known to be less biologically responsive. Semi-synthetic polymers allow for the combination of natural and synthetic properties to create new materials with fewer drawbacks [64,65]. Table 1 summarizes the most recent studies on polymer scaffold materials for corneal endothelium engineering. 

### 4.3. Natural and Semi-Synthetic Polymers

Collagen is a widely studied natural polymer for scaffold innovation, as it is a predominant protein in the cornea with great biocompatibility and biodegradability, as well as low adverse immune effects. However, collagen is known to lack important mechanical properties, such as toughness and elasticity. Mimura et al. (2004) first found great potential in their study by transplanting collagen sheets in a rabbit model [66], and Vazquez et al. (2016) tested human bone derived type 1 collagen on rabbit models with satisfactory results [67]. Many studies have attempted to innovate collagen scaffolds with different variations. Levis et al. developed a plastic-compressed type 1 collagen gel, which was tested in vivo on a rabbit model by Xiao et al. with promising results of a mechanically stronger, biocompatible, but semi-transparent scaffold [68,69].

Gelatin is another promising and frequently used natural material in medicine, obtained through hydrolyzing collagen [70]. It has great biocompatibility, biodegradability, transparency, elasticity, and permeability [71]. Niu et al. (2014) modified gelatin scaffolds with heparin to yield scaffolds with better growth factor binding to promote CEC growth after the implantation of the scaffold and tested them in vitro and in vivo with rabbit models [72]. Furthermore, a semi-synthetic gelatin-based scaffold with a curcumin and lipid-poly(lactic-co-glycolic acid) was developed by Li et al. (2021). Their in vitro study showed good cell proliferation and growth with antioxidative, anti-inflammatory, and anti-angiogenic effects [73]. Gelatin methacrylate (GelMA) is another semi-synthetic gelatin-based polymer with good biodegradability and biocompatibility, currently studied for corneal regeneration. Yan et al. studied GelMA in different concentrations in vitro and found that lower-concentration gels had better hydrophilicity and optical properties, while higher-concentration gels had better mechanical properties and cell differentiation for stroma transplantation [74]. A variation of GeIMA with silk nanofibrils was studied by Farasatkia et al. The variation yielded promising results, with 30SNF/70GeIMA being the most promising ratio needed for a corneal regeneration [75]. This finding will also be mentioned in the topology section in relation to its positive impact on scaffold transparency.

Silk fibroin is a natural protein polymer with great biocompatibility and tensile strength. It has been studied both as a complement to toughen collagen [76] and as a stand-alone film [77]. Silk fibroin has also been electrospun with poly(L-lactic acid-co-ε-caprolactone) at different blended ratios of semi-synthetic scaffolds with good results for biocompatibility [78]. Vazquez et al. studied a silk fibroin film obtained from Bombyx mori cocoons. They cultivated both human and rabbit corneal endothelial cells on the films and transplanted the rabbit corneal endothelial graft in vivo in a rabbit model by DMEK. The films were able to support the growth of both human and rabbit CECs, and the transplanted tissue was able to properly integrate into the cornea and restore transparency [79]. Recent studies experimented with variations of silk fibroin. Ramachandran et al. studied non-mulberry varieties derived from Philosamia ricini (PR), Antheraea assamensise (AA), and the more commonly used Bombyx mori (BM) in vitro. PR and AA demonstrated better cell adherence, structure, and elasticity than BM, and all three had a >90% light transmittance and a refractive index similar to that of the human cornea [80]. Kim et al. (2021) used curcumin to enhance silk fibroin in vitro and obtained enhanced cell proliferation with good hydrophilicity and transparency [81].

Hyaluronic acid (HA) is a glycosaminoglycan with diverse cell regulating functions, making it an interesting substrate material. Several modifications of HA were studied, such as HA hydrogels crosslinked by carbodiimide, which improved their biocompatibility but posed a problem to intraocular pressure due to their high density [82]. Porous HA hydrogels were also studied in vivo to counter this issue and demonstrated therapeutic potential but failed to achieve full tissue repair [83]. More recently, Nguyen et al. tested the effects of oxidized aldehyde HA on gelatin microcarriers in vitro and in vivo on injured rabbit corneas that showed great biocompatibility, improved cell adhesion, and corneal repair, showing great results for stromal regeneration [84]. Another in vitro study crosslinked HA with bacterial nanocellulose (BNC), another natural polymer. The addition of HA increased BNC’s light transmittance by 40% with great biocompatibility, showing great potential for this composite material as an application in corneal transplantation [85]. A new promising approach was introduced by Zhao et al., incorporating superparamagnetic nanoparticles into HA to make magnetic HA gels. This magnetic system allows for precise cell delivery, and more studies will be conducted to evaluate its transparency, thickness, and therapeutic function [86].

Chitosan is a natural polysaccharide with good biocompatibility and biodegradability. Studies have combined it with other materials such as collagen [87], poly(ethylene glycol) [88], and polycaprolactone [89]. Tayebi et al. developed a semi-synthetic chitosan and polycaprolactone (PCL) scaffold with chitosan nanoparticles, which improved biocompatibility and surface properties while preserving transparency [90].

Agarose is a natural polysaccharide that has great biocompatibility and mechanical properties but lacks proper cell adhesion. Seow et al. modified ultra-thin agarose scaffolds with different attachment signals (GRGD, lysine, poly lysine, and fish-derived gelatin) and tested them in vitro with rabbit CECs. The fish-derived gelatin/agarose had the best results for cell attachment and viability and had a >96% light transmittance [91].

Alginate is a natural polymer with good biocompatibility and controllable physical properties. Recently, Song et al. (2024) tested in vitro an alginate hydrogel with an integrated human fibroblast-derived ECM. The scaffold demonstrated good surface properties and an ideal microenvironment for cell adhesion and growth [92].

### 4.4. Synthetic Polymers 

Several synthetic polymers and composites have been studied in the last two decades in corneal endothelium engineering, such as polyvinylidene fluoride [52], poly(N-isopropylacrylamide) [93], poly(glycerol sebacate)/polycaprolactone (PCL) [94], poly(methyl-methacrylate) (PMMA) [95], collagen-immobilized poly(ethylene glycol) (PEG)/poly(2-hydroxyethyl methacrylate) [96], poly(DL-lactic acid-co-glycolic acid) (PLGA)/PEG [97], and collagen-coated PLGA [98].

Notably, Ozcelik et al. (2014) tested a PEG hydrogel with PLC that yielded a > 98% transparency with regeneration properties and biodegradability [99]. PEG is a hydrophilic synthetic polymer that has been approved by the Food and Drug Administration (FDA), and polycaprolactone (PLC) is a hydrophobic synthetic polymer known for its biocompatibility and biodegradability. Himmler et al. (2021) further studied PLC by electrospinning four types of scaffolds, namely PCL, PCL with chitosan, PCL with collagen, and PCL with gelatin. The last two yielded the best results in cell viability, and all four were non-cytotoxic [100]. Kruse et al. produced electrospun PMMA, PLGA, and PCL scaffolds and cultured human CECs on these three scaffolds. PMMA is a synthetic non-degradable polymer known for its good biocompatibility, and PLGA is a synthetic copolymer with great biocompatibility and biodegradability that has also been approved by the FDA for many biomedical uses. PMMA had the best light transmission but showed cytotoxicity. Only the PLGA scaffold was able to maintain proper cell morphology, and both PLGA and PCL showed good cell viability and biocompatibility [95].

Recent studies have been conducted on poly-ε-lysine (pεK), a synthetic, hydrophilic, biocompatible, non-toxic peptide. Kennedy et al. crosslinked a pεK hydrogel with octanedioic acid and tested the scaffold in vitro on human CECs, as well as primary porcine CECs [101]. Lace et al. tested a variation of pεK with regulatable characteristics by modifying the percentage of the bis-carboxylic acid crosslinker for corneal regeneration [102]. Both studies presented pεK as a promising candidate.

**Table 1:** Summary of recent studies on polymer scaffold materials for corneal endothelium engineering (up to 5 years).

Material	Polymer Type	Study Type	Results	Year	Reference
Curcumin-loaded lipid-PLGA hybrid microparticles/gelatin (Cur@MP/gelatin)	Semi-synthetic	In vitro culture of rabbit CECs	Cur@MP demonstrated anti-inflammatory, anti-oxidative, and anti-angiogenic properties. Cur@MP/gelatin scaffold supported formation of CEC monolayer [103,104].	2021	[73]
Non-mulberry silk fibroin	Natural	In vitro culture of human CECs	Good tensile strength, >95 light transmittance and refractive index. Better cell adhesion and monolayer formation on non-mulberry variations.	2020	[80]
Curcumin-enhanced silk fibroin	Natural	In vitro culture of rabbit CECs	Rough surface of the scaffold promoted cell adhesion and proliferation. Good hydrophilicity and transparency.	2021	[81]
Injectable magnetic hyaluronic acid gel	Natural	In vitro and in vivo assessment in a rabbit model	Precise cell delivery and retention in vivo.	2024	[86]
Chitosan and polycaprolactone scaffold with chitosan nanoparticles	Semi-synthetic	In vitro culture of human CECs	Improved biocompatibility and surface properties with maintained transparency.	2021	[90]
Agarose modified with GRGD, lysine, poly lysine, and fish-derived gelatin	Natural	In vitro testing with human and rabbit CECs	Fish-derived gelatin scaffolds had the best results with viable rabbit CECs for 4 weeks, better cell attachment, and >96% transparency.	2019	[91]
Alginate hydrogel with human fibroblast-derived ECM	Natural	In vitro transplantation of human CECs into a decellularized porcine cornea	Suitable microenvironment for cell attachment and growth.	2024	[92]
Polycaprolactone (PCL), PCL/collagen, PCL/gelatin, and PCL/chitosan	Synthetic and semi-synthetic	In vitro testing with human CECs	PCL/collagen and PCL/gelatin yielded the best cell viability.	2021	[100]
Poly-ε-lysine hydrogels	Synthetic	In vitro cell culture of human and porcine CECs	Good attachment and growth of human CECs but detachment of porcine CECs.	2019	[101]

## 5. Micro- and Nano-Topological Morphologies

### 5.1. Importance of Surface Morphology in Cell Behavior 

Surface morphology plays a crucial role in tissue engineering. It is well established that CECs, like all cells, are sensitive to their environment, and that the topographical features of the ECM support cell adhesion, arrangement, and organization while maintaining their phenotype and function [25,105,106].

Employing DM-like microtopography has a potential so strong that it has succeeded in differentiating human mesenchymal stem cells into CEC-like cells in vitro in a study conducted by Gutermuth et al. (2019) [107]. More recently, Özgen Öztürk-Öncel et al. (2021) have shown that this approach can also improve in vitro substrate properties of polydimethylsiloxane (PDMS) for corneal endothelium tissue engineering. PDMS was first functionalized with collagen IV and hyaluronic acid, two essential components of the ECM. Subsequently, rose petal topographical features, which have been shown to be similar to those of the corneal endothelium, primarily due to their hexagonal cell shapes and cell density (≈2000 cells/mm^2^), were incorporated onto the surface. This creation of an ECM-like microenvironment significantly increased bovine CEC proliferation and preservation of the CEC-specific phenotype [108]. There have been numerous other attempts to mimic the three-dimensional physical configuration of the ECM in corneal endothelial tissue engineering with very promising results [109,110,111,112]. To learn more about them, please refer to Table 2.

**Figure 5:**
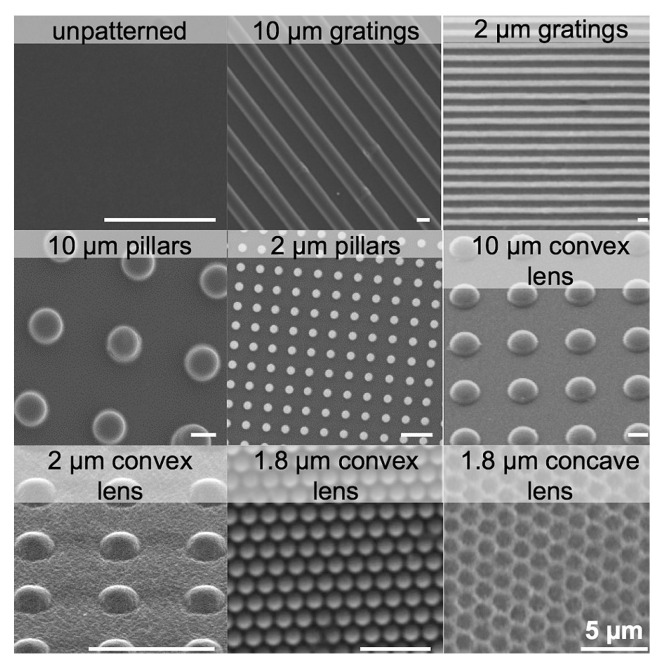
Topological features with different shapes and sizes that can be used for surface patterning [113]. Reprinted with permission from ref. [113]. Copyright 2016 Elsevier.

The following section will explore advancements in micro- and nano-topological morphologies with favorable results, as well as their impact on cell behavior. As a foundation, Figure 5 [113] illustrates various predesigned patterns with different shapes and sizes that are commonly used in scaffold design.

First, surface morphologies, in combination with material selection, can be employed to enhance scaffold transparency. For instance, highly transparent hybrid films were created by Farasatkia et al. (2021) by combining silk nanofibrils and GelMA. The films also exhibited favorable mechanical properties, as well as optimal cell attachment, spreading, and proliferation [75]. Additionally, in a study by Xiong et al. (2019), microgrooved collagen-based scaffolds have demonstrated optical clarity comparable to the natural human cornea, as well as excellent biodegradability. The scaffolds also boosted the cell alignment index from 20% to 60% and accelerated wound healing by inhibiting fibrosis [114].

As for protein adsorption, it refers to the process where proteins from a fluid (such as blood or cell culture media) adhere to a material. This initial protein adhesion is often a nonspecific process that can lead to the interaction of undesired particles with a particular surface. This phenomenon can hinder the selective growth of cells, such as CECs, when designing scaffolds. This is why materials that are non-adhesive like PEG are widely used [115,116]. However, surface topographies are also vital in ensuring materials are inert and resistant to unspecific protein adhesion. Certain surface coatings, such as self-assembled monolayers (SAMs), which are covalent coatings of oligo(ethylene glycol)s [117,118], as well as “brush”-like patterns [119] and star shapes on PEG hydrogels [120], are found to prevent unspecific protein adsorption and keep the surface non-fouling. Other surface modifications proven to create bioinert materials are phosphorylcholine, poly(ethylene glycol), and poly(ethylene oxide) coatings, as well as hyaluronic acid (HA) crosslinking [121]. However, these are chemical surface treatments rather than topographical features.

**Figure 6:**
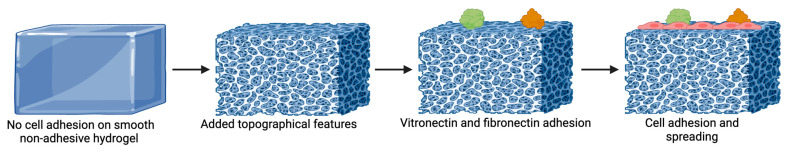
Enhanced protein adsorption and cell adhesion on micropatterned hydrogels. Created with BioRender.com.

Nevertheless, it is important to note that patterns that are able to enhance the adsorption of specific proteins like fibronectin (FN) and vitronectin (VN) can be highly beneficial. These proteins, which are a part of the ECM, provide specific binding sites that are known to promote cell adhesion. As illustrated in Figure 6, in intrinsically non-adhesive materials like Acr-sP(EO*-stat-*PO) hydrogels and starPEG hydrogels, micropatterning induces increased vitronectin (VN) adsorption and consequently, strong cell adhesion, alignment, and spreading [122,123].

Another general guideline for enhancing cell adhesion is to incorporate features smaller than 2–5 nanometers, known as subcellular patterns. Whether they are nanoparticles [124,125], nanotubes [126,127], or nanofibers [128,129,130], these are more effective than larger micron-scale features in maintaining the human CECs’ phenotype. This is because they more closely mimic the nanosized biomolecules and cells that interact with the ECM in native tissues.

In addition to the ones previously discussed, other crucial properties that are influenced by topography that must be considered when designing scaffolds are cell proliferation and differentiation and the wetting state of a material, as well as bacterial adhesion, given that patients can suffer from infections or even death if pathogenic bacteria attach to implants.

Significant research findings on optimizing the antibacterial performance of surface topographies are detailed on Table 3. Some general conclusions and principles to be retained are that smooth surfaces are generally less effective in preventing bacterial adhesion compared to textured or rough surfaces. Furthermore, smaller features tend to inhibit bacterial attachment more effectively than larger features. For example, nanopores with pore diameters of 15–25 nm are more effective than larger pores (e.g., 50–100 nm) at reducing bacterial adhesion and biofilm formation [131]. This is because ensuring that dimensions are smaller than bacterial cells will prevent bacteria from attaching and entering crevices. Moreover, higher spacing diameters (e.g., 400 nm) result in less bacterial adhesion compared to smaller spacing (e.g., 50 nm) [132]. Concerning the shapes of topographic features, X-shaped pillars or other similar complex geometries proved to be the most effective at preventing bacterial adhesion by providing minimal contact points for bacteria [133]. Other general principles are that the height of the topographical patterns should be higher than the length of bacteria flagella (at least 10 μm) to prevent them from entering the groove [134], and that to prevent biofilm from developing, plateau areas should be less than 400 μm², since it has been shown that bacteria like E.coli significantly adhere to plateaus only when they are larger than 20 μm × 20 μm [135].

Many studies also demonstrate that surface topography has a significant impact on the wetting state of a biomaterial in addition to its hydrophobic and hydrophilic properties [136,137,138,139,140]. As previously discussed, hydrophobicity is a crucial aspect of biomaterials that can optimize a scaffold for various applications. Here are some common findings of recent studies regarding how surface topography influences hydrophobicity. First, surface hydrophobicity can be enhanced by increasing the height of nanoscale pillars, but only if the initial hydrophobicity is above a certain threshold. If the surface is already somewhat hydrophobic (contact angle > 93.13°), making the pillars taller (from 2.82 nm to 3.76 nm) makes the surface even more hydrophobic (Cassie–Baxter state). However, if the surface is not very hydrophobic (contact angle < 85.1°), increasing the height of the pillars has no effect on changing the wetting state, and the surface remains in a state where water fills the grooves (Wenzel state) [141]. Furthermore, adding micro-scale grating patterns (e.g., 2 μm) to hydrogels, as well as biomimetic topographies like those mimicking lotus leaves, can increase hydrophobicity, resulting in enhanced cell adhesion and spreading [113,142]. Lastly, larger, rectangular pillar structures can also significantly increase hydrophobicity, even when tested with different liquids [143].

The following table summarizes the key findings and applications of the micro- and nano-topological morphologies from the various studies previously mentioned.

**Table 2:** Key findings, applications, and impact of micro- and nano-topological morphologies on various properties.

Material	Topographical Property Features	Cell Type Affected	Effect of Added Topographical Feature	Key Applications	Fabrication Technique Used	Key	Reference
Silicone and collagen	Hexagonal structures, 1.52 to 2.02 µm depth, 10–20 µm width	Human mesenchymal stem cells (hMSCs)	Differentiation of hMSCs into corneal endothelial-like cells	Corneal endothelium tissue engineering, potential autologous stem cell therapy	Two-photon lithography	2019	[107]
Tissue culture polystyrene (TCPS)	1 μm pillars, 1 μm wells, and 250 nm pillars; FNC coating containing fibronectin, collagen I, and albumin	Human corneal endothelial cells (HCECs)	Enhanced proliferation, maintenance of functional markers like ZO-1 and Na+/K+-ATPase	Corneal endothelium tissue engineering, cell therapy, and drug screening	Heat embossing	2015	[109]
PDMS (polydimethylsiloxane)	1 μm pillars, 1 μm wells, 250 nm pillars, and 250 nm wells	Bovine corneal endothelial cells (BCECs)	Enhanced cell density on pillars, maintenance of functional markers like Na+/K+-ATPase	Corneal endothelium tissue engineering and drug screening	Soft lithography	2012	[110]
PDMS (polydimethylsiloxane)	Nanopillars: 250 nm diameter, 250 nm height; micropillars: 1 µm diameter, 1 µm height; FNC coating containing fibronectin, collagen I, laminin, and chondroitin sulfate	Human corneal endothelial cell line B4G12 (HCEC-B4G12)	Increased cell proliferation; improved Na+/K+-ATPase and ZO-1 expression	Corneal endothelium tissue engineering	Soft lithography	2014	[111]
Gelatin methacrylate (GelMA)	1 μm pillars with 6 μm spacing (1 × 6 μmpS pillars); 250 nm pillars	Human corneal endothelial cells (HCECs)	Enhanced cell adhesion and mechanical strength, customizable degradation rates, increased amount of ZO-1 expression for 1 × 6 μmpS pillars	Corneal endothelium tissue engineering	-Hybrid crosslinking method (which combines physical crosslinking followed by UV crosslinking to improve the material’s mechanical strength) -PET stamp-based nano-molding for high-resolution patterns	2017	[112]
Silk nanofibrils (SNF) and gelatin methacryloyl (GelMA)	Nanoscale fibrillar structures with 30/70 volume ratio of SNF/GelMA	Human corneal stromal cells	Enhanced transparency, mechanical strength, cell attachment, spreading, and proliferation with customizable degradation rates	Cornea regeneration	UV crosslinking for GelMA; calcium chloride–formic acid dissolution and stirring for nanofibril formation. Casting followed by UV crosslinking for fabrication of SNF/GelMA hybrid films.	2021	[75]
Acrylated star-shaped poly(ethylene oxide-stat-propylene oxide) (Acr-sP(EO-stat-PO)) hydrogels	Micrometer-sized surface patterns (posts and grooves). Groove width: 10 μm, depth: 5 μm (grooves separated by 20 μm space). Wider pattern: 25 μm wide grooves, 10 μm depth (separated by 25 μm spaces)	Mouse fibroblast cell line (L929) and human mesenchymal stem cells (hMSC)	Induced vitronectin (VN) adsorption and strong cell adhesion, alignment, and spreading	Use topographic patterning to promote cell adhesion even on non-adhesive materials without additional surface chemistry modifications	UV-based imprinting	2011	[122]
Star-shaped poly (ethylene glycol)	Posts: 3 µm diameter, 3 µm height; lines: 5–50 µm spacing, 5–50 µm width, 5 µm depth	Mouse fibroblast (L929)	Enhanced cell adhesion and spreading. Posts: cells spread, wrapped around posts. Lines: cells aligned along grooves, best alignment in 5–10 µm grooves.	Use topographic patterning to promote cell adhesion even on non-adhesive materials without additional surface chemistry modifications	Nanoimprinting and replica molding	2009	[123]
Poly(vinyl alcohol) (PVA) hydrogel	Gratings, pillars, convex lens, concave lens (gratings: 250 nm, 10 μm, 2 μm; pillars: 10 μm, 2 μm; convex lens: 10 μm, 2 μm, 1.8 μm; concave lens: 1.8 μm)	Human endothelial cells (EA.hy926)	The 2 μm gratings on PVA hydrogels were found to increase hydrophobicity and were the most effective in promoting endothelial cell adhesion and density. Convex and concave lenses also performed well but were slightly less effective than gratings. Pillars were moderately effective and were the least optimal.	Corneal endothelium tissue engineering	Casting method (for the creation of micro-sized features), nanoimprint lithography (for the creation of nano-sized features), and nitrogen plasma modification	2016	[113]
PHEMA hydrogels	Lotus leaf topography (3 ± 1 µm height, 9 ± 2 µm width)	Human corneal epithelial cells (HCE-T)	Enhanced cell adhesion and proliferation, increased hydrophobicity with static contact angle of 86 ± 2°, and the presence of trapped air pockets.	Tissue engineering, especially in applications requiring enhanced cell adhesion and hydrophobic surfaces	Polymerization in mold-nanoimprint lithography (PIM-NIL), which involves the use of Teflon AF molds to capture the hierarchical structure of the lotus leaf, both at the micro- and nanoscale, and then polymerizing PHEMA within the mold to create a structured hydrogel.	2016	[142]
PHEMA (poly(hydroxyethyl methacrylate))	Pillar structures (aspect ratio up to 100:1, 30 µm diameter, 1 mm height, 50–500 µm spacing)	Not specified	Microstructured surfaces showed higher contact angles compared to smooth surfaces, indicating increased hydrophobicity	Biomimetic surfaces and hydrophobic surface design	Combination of molding and radical polymerization	2005	[143]
Collagen films	Groove widths of 25 µm, 50 µm, and 100 µm, with a depth of 50 µm and a ridge width of 200 µm	Rabbit corneal epithelial cells (CECs) and keratocytes	Swelling capacity and optical clarity comparable to natural cornea, similar degradation rate to unpatterned films (14 h), significant cell alignment along grooves (alignment index 20% to 60%), normal exponential cell growth (slightly slower on wider grooves), accelerated wound healing with narrower grooves, and inhibition of keratocyte transformation into myofibroblasts (reduced CTGF, aSMA, COL1A1 gene expression).	Design surfaces that promote cell alignment, guide direction migration of cells, accelerate wound healing, and inhibit fibrosis. Epithelialization of corneal epithelial cells.	Combination of soft lithography and solvent casting	2019	[114]
Polydimethylsiloxane (PDMS)	Rose-petal-topography-mimicked surface. Microgroove mean depths of 12.9 µm (red rose) and 6.6 µm (white rose). White rose petal patterned PDMS exhibited hexagonal patterns similar to CEC.	Bovine corneal endothelial cells (BCE C/D-1b)	Collagen IV-functionalized PDMS (PDMS-C4) significantly enhanced CEC proliferation, but white-rose-patterned PDMS-C4 (PDMS-C4-R) provided the highest proliferation rate and cell density. PDMS-C4-R also maintained CEC-specific phenotype.	Corneal endothelium tissue engineering	Soft lithography, followed by functionalization with collagen IV and hyaluronic acid to enhance cell attachment and proliferation.	2021	[108]
Gold with SAMs of oligo(ethylene oxide)	Self-assembled monolayers (SAMs) with variable chain lengths	Proteins (e.g., fibrinogen, lysozyme)	Prevention of nonspecific protein adsorption	Design of non-fouling surfaces for biomedical applications	Adsorption of alkanethiols onto gold surfaces using ethanol solutions, leading to the creation of monolayers with oligo(ethylene oxide) chains	1993	[118]
Glass, silicon, and titanium panes	Ultrathin film (30 +/- 5 nm) of reactive star-shaped poly(ethylene glycol) prepolymers (star PEG).	Human dermal fibroblasts (HDFs), sarcoma osteogenic cells (SaOS-2), human mesenchymal stem cells (hMSCs)	Prevention of unspecific protein adsorption. Promotion of specific cell adhesion and proliferation while preserving normal differentiation process.	Non-fouling implant coatings that promote cell adhesion and proliferation	Not mentioned	1991	[119]
	Linear RGD peptide (gRGDsc)-modified star PEG coatings.

**Table 3:** Significant research findings on optimizing the antibacterial performance of surface topographies.

Material	Topographical Property Features	Cell Type Affected	Effect of Added Topographical Feature	Key	Reference
Polydimethylsiloxane (PDMS)	10 µm pitch (square and circular features), 5 µm pitch (parallel channels); heights: 21.1 nm and 117 nm	*Staphylococcus* epidermidis, *Escherichia coli*, Bacillus subtilis	Significant reduction in bacterial adhesion (30–45%) [103,104]	2014	[144]
Poly(dimethylsiloxan) (PDMS)	10 µm tall square features, varied side lengths (2, 5, 10, 15, 20, 30, 40, 50, or 100 µm) and distances (5, 10, 15, or 20 µm) between features	*Escherichia coli* (*E. coli*) RP437/pRSH103	*E. coli* formed biofilms mainly in valleys between features, with significant formation on protrusions only when plateaus were at least 20 µm × 20 µm (face-up) or 40 µm × 40 µm (face-down), indicating a size threshold for adhesion. Motility increased adhesion.	2011	[135]
Poly(dimethylsiloxan) (PDMS)	Hummock patterns (2.7 µm height, 3 µm diameter, 440 nm trenches)	*E. coli* (wild type and various mutants)	Wild-type *E. coli* showed increased adhesion over time, particularly with flagella exploring crevices. Mutants lacking flagella or motility showed reduced adhesion. The structured surfaces initially reduced adhesion, but wetting altered this.	2013	[145]
Polydimethylsiloxane (PDMS)	Line patterns (width: 5 µm, 10 µm, 20 µm; height: 5 µm; inter-pattern distance: 3 µm, 5 µm, 10 µm, 20 µm) and hexagon patterns (height: 10 µm, side length: 2 µm, 5 µm, 10 µm, 15 µm, 20 µm; inter-pattern distance: 2 µm, 5 µm, 10 µm, 15 µm, 20 µm).	*Escherichia coli*	Narrow patterns (5 µm) reduced cell cluster formation and biofilm formation; cells oriented perpendicularly on narrow patterns; flagella played a key role in cell orientation. Hexagon patterns with side length of 15 µm and inter-pattern distance of 2 µm reduced biofilm formation by 83.6% compared to flat PDMS.	2016	[134]
Nanocrystalline nickel	Solid core pillars (1000 nm diameter, height-to-diameter ratio 1.5),	*Staphylococcus aureu*s	The X-shaped pillars exhibited the lowest success rate for bacterial adhesion, making them the most effective for preventing bacterial colonization. Mushroom-shaped nanostructures showed the highest bacterial attachment, making them the least effective.	2014	[133]
	hollow pillars (1000 nm outer diameter, 840 nm inner diameter, height-to-diameter ratio 1.5),				
	C-shaped pillars (1000 nm outer diameter, 760 nm inner diameter, height-to-diameter ratio 1.5),				
	X-shaped pillars (1000 nm outer diameter, wall thickness 300 nm, height-to-diameter ratio 1.5), and				
	mushroom-shaped nanostructures (stem diameter 220 nm, cap diameter 1400 nm).				
Aluminum oxide	Nanopores (15, 25, 50, 100 nm pore diameters)	*Escherichia coli* (*E. coli*), Listeria innocua (L. innocua)	Nanopores of 15 and 25 nm diameters significantly reduced bacterial attachment and biofilm formation. The 50 and 100 nm pores showed higher levels of bacterial adhesion and biofilm formation.	2014	[131]
Polyethylene terephthalate (PET)	Nanopillar arrays (diameter: ~250 nm, height: 1000 nm, interpillar spacing: 50 nm, 200 nm, 400 nm)	*Escherichia coli* (*E. coli*), *Staphylococcus aureus* (*S. aureus*), *Helicobacter pylori* (*H. pylori*)	The 50 nm spacing promoted adhesion, 200 nm spacing reduced adhesion compared to 50 nm, and 400 nm spacing resulted in the least adhesion; morphological changes in bacteria observed.	2015	[132]

### 5.2. Techniques for Creating Micro- and Nanostructures

The creation of biocompatible scaffolds requires mimicking biological structures at the macro (organ), micro (cell), and nano (molecular) scales [146]. There are various techniques available to fabricate such micro- and nanostructures. This section will explore these techniques and their applications in tissue engineering.

Techniques that create micro- and submicron scale structures include micromachining, photolithography, metal deposition, electrospinning, wet and dry etching, thin film growth, and 3D bioprinting [147,148,149]. Lithography, deposition, and etching are generally not suitable on their own for creating scaffolds that mimic biological structures, but more so for patterning materials like silicon and glass. This is why those methods often need to be combined with other techniques, such as replica molding and embossing for medical purposes [150]. Replica molding involves creating a high-resolution master and replicating its features onto a new material. This replication can be performed using techniques such as soft lithography, nanoimprint lithography, and micro-injection molding. These methods are advantageous due to their low cost and suitability for large-scale production [150,151]. For example, Limongi et al. (2015) produced polycaprolactone (PCL) pillared scaffolds by first creating a high-resolution silicon master using lithography to achieve features smaller than 50 nm. Then, micromolding with hot embossing and injection molding machines was used to press the material. After solidification and detachment, the final scaffold was obtained [152]. Other excellent techniques that create nanofeatures include high-resolution methods such as two-photon lithography, electron beam lithography, and focused ion beam lithography. Those techniques are described in Table 3.

### 5.3. Combining Techniques for Enhanced Scaffold Fabrication

**Figure 7:**
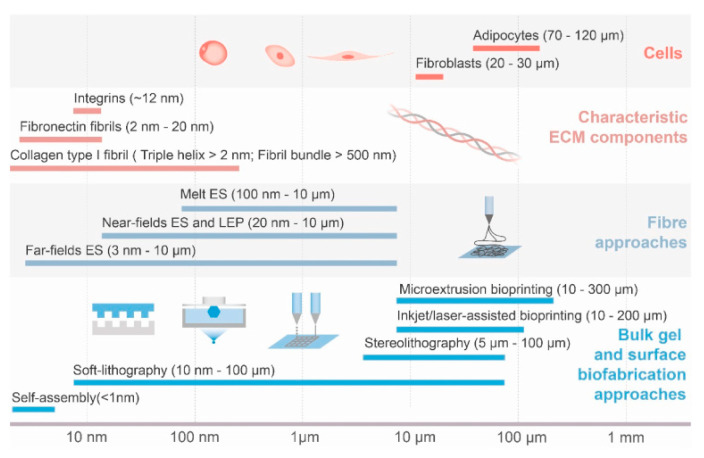
Length scale bar illustrating featured resolutions of various biofabrication techniques in comparison with geometric sizes of representative cells and tissues [154]. Reprinted from ref. [154], licensed under CC BY 4.0 [https://creativecommons.org/licenses/by/4.0/]. Accessed on 3 August 2024. “ES” stands for electrospinning and “LEP” stands for low-voltage electrospinning patterning.

Combining complementary techniques has led to significant advancements by helping to eliminate the limitations of individual methods, thus achieving the resolution and complexity needed to mimic natural tissues and enhancing the properties of the scaffolds [153]. It also helps to cover all length scales of the ECM components that are intended to be replicated [154]. Figure 7 [154] illustrates the length of various ECM components and the resolutions/sizes of features that different biofabrication approaches can provide.

A few examples include combining solution electrospinning with extrusion-based printing to enhance cell entrapment, adhesion, and proliferation, as well as combining melt electro-writing and extrusion-based bioprinting to increase design freedom, mechanical stability, and tissue mimicry, while preserving cell viability and differentiation [155,156,157,158]. Another example is SMART (substrate modification and replication by thermoforming) technology, which combines various polymer modification methods with microthermoforming as a post-processing method. It is a process that shapes polymer films by heating them to a pliable thermoelastic state and then forms them into desired shapes. This technique, described by Giselbrecht et al. (2006), does not involve any melting, unlike typical micromolding methods, which helps preserve any modifications made to the polymer prior to formation [159]. This technique has shown potential for tissue engineering applications. For instance, it was used by Truckenmüller et al. (2011) to shape thin polylactic acid films that have been prepatterned with thermal nanoimprinting into microcontainer arrays shaped like a spherical calotte (dome-like shape) that transitions into a hexagonal shape at the top edges. Testing showed the successful adhesion and alignment of cells along microgrooves, indicating the potential for these substrates in tissue engineering [160]. The combination of low-voltage electrospinning patterning (LEP) with 3D bioprinting to create cell culture devices that show excellent cell attachment, viability, differentiation, and fibronectin secretion [161], as well as combining continuous chaotic printing and electrospinning, known as chaotic electrospinning [162], have also exhibited promising results. Although it is mostly used to create electrodes for supercapacitors, chaotic electrospinning, with its capacity to fabricate polymer fibers with complex multilayered nanostructures at a large-scale production level, could be highly relevant for developing complex scaffolds in corneal tissue engineering. Moreover, photolithography and two-photon polymerization (TPP) were combined for the first time by Lin et al. (2018) to create master molds for soft lithography, which resulted in the efficient fabrication of high-resolution microstructures [163].

### 5.4. Soft Litography and Organ-on-a-Chip Designs

Soft lithography is a promising nanofabrication technique with several advantages, as seen in Table 4. A unique element in this technique is its role in the creation of microfluidic technology, which has paved the way for organ-on-a-chip designs [154]. An organ-on-a-chip is a device that has been widely used to mimic the structure and physiological functions of human organs in vitro and in vivo, such as the lungs [164], the heart [165], the liver [166], the kidneys [167], the intestines [168], the brain [169], and the vasculature [170]. Recent advances have also been made in corneal organ-on-a-chip designs [171,172,173], and like in the other fields, they show great promise in nano-medicine, disease modeling, and drug development [174]. Cornea-on-a-chip (CoC) microfluidic platforms have contributed to the fabrication of several physiologically relevant corneal microstructures [175], including corneal endothelium- and epithelium-related models [176,177,178]. These CoC platforms are primarily used right now for the preclinical evaluation of drugs. Nonetheless, although this is not yet a widespread or standard practice, their capability of accurately replicating in vivo characteristics could also be valuable in the preclinical testing of scaffolds. By observing how corneal endothelial cells grow, adhere, and function on scaffolds within the simulated corneal environment, researchers could potentially refine scaffold design before progressing to further testing, such as in vivo studies or clinical trials. Utilizing these advanced in vitro experimental models could also deepen our understanding of the various biological processes behind endothelial cell proliferation and topological morphology, which is necessary for successful clinical translation, as mentioned in Section 7.

### 5.5. Electrospinning

Electrospinning is another promising emerging technology. Its key features include its high productivity, low cost, simplicity, and versatility. Its ability to create ECM-mimicking 3D fibrous structures with excellent fiber alignment helps preserve the natural cell phenotype and enhance migration and proliferation [179,180]. Utilizing electrospinning to fabricate scaffolds for human corneal endothelial cells has proven to be effective in a wealth of research. For instance, Kruse et al. (2017) highlighted the suitability of electrospun PLGA and PCL scaffolds, which demonstrated high biocompatibility and supported the growth and viability of hCECs [95]. Other examples of successful electrospun scaffolds for corneal regeneration include the fabrication of a matrix made of electrospun GelMA fibers and p(HEMA) [181] of an electrospun gelatin nanofiber (gelNF) membrane crosslinked with glutaraldehyde (GA) [182], of electrospun non-woven with varied fiber orientations and topographies [183], and of electrospun nanofibrous gelatin mats crosslinked with genipin [184]. These scaffolds demonstrated desirable outcomes such as mechanical strength, transparency, cell adhesion and growth, biocompatibility, and the preservation of the cell phenotype, with all of them exhibiting several or more of these properties.

It is also important to know about the limitations of electrospinning to determine how to overcome them. Its first limitation is its inability to create a full-thickness corneal scaffold [185]. However, this is less relevant to our work, as our primary focus is on scaffolds for the corneal endothelium. Another limitation of electrospinning is the low light transmission that results from the slim interfiber spacing of synthetic polymer electrospun fibers [185]. However, treatments such as helium–oxygen plasma for PCL fibers [186], laser perforation for PLGA fibers [187], and embedding poly(2-hydroxymethyl methacrylate) in gelatin fibers [181] have all significantly improved light transmittance to suitable levels for corneal scaffolds. Lastly, combining natural biopolymers and synthetic polymers can optimize the overall performance of electrospun scaffolds by ensuring the fibers are strong enough to withstand the electrospinning process without adversely affecting the phenotype of the cells [188,189]. For example, blending gelatin with PCL increased tensile stress tenfold, from 0.11 to 1.23 MPa [190].

The following table summarizes some high-yield nanofabrication techniques for scaffolds with their pros, cons, and applications.

**Table 4:** Overview of nanofabrication techniques for scaffold engineering.

Techniques	Description	Advantages	Challenges	Applications	Reference
Soft lithography	Uses elastomeric stamps (most notably PDMS) to transfer patterns from a master template onto another material	Flexible (can be used for patterning a variety of materials) pillars, valves, and stretchable membranes. Capable of creating patterns on flexible and large-area substrates Reusable PDMS stamps Cost-effective High reproducibility High lateral resolution Can form temporary and adaptable contacts with complex geometries Suitable for modeling three-dimensional in vivo environments with few complications	Cannot reliably create features smaller than 100 nm. Transferring 2D resist patterns to a functional layer for 3D fabrication is as an arduous process.	Tissue engineering Microfluidic devices for cell cultivation and simulating tissue microenvironments Semiconductor manufacturing	[174,191,192,193,194]
Electron-beam lithography (EBL)	Uses a focused beam of electrons to create patterns on a surface covered with an electron-sensitive resist	Rapid prototyping High resolution down to 5 nm Versatile for substrates No need for templates Can pattern nonplanar and irregular surfaces Can be combined with other techniques like cryostases evaporation systems and metal deposition for enhanced capabilities Can expose a thick resist without ion contamination	Low fabrication speed Costly Requires complex proximity correction processes to compensate for light distortion and scattering of particles Challenging to apply for large-area patterning and on curved surfaces	Creation of detailed cellular scaffolds Fabrication of nanoscale biosensors Development of lab-on-a-chip devices Development of porous and fragile membranes for biological applications	[191,195,196]
Focused ion beam lithography (FIB)	Utilizes a focused beam of ions, typically gallium, to directly write or mill patterns onto a substrate	Rapid prototyping High resolution down to 5 nm Significant larger depth of focus compared to EBL Very small scattering of ions in a resist layer Can pattern on highly corrugated surfaces Can process a wide range of materials No need for masks Capable of fabricating suspended nanostructure	Achievable resolution lower than EBL Low fabrication speed Costly Potential damage to the substrate due to ion implantation	Fabrication of nanoscale biosensors Creation of microfluidic devices for cell studies Modification of biomaterials for tissue engineering Development of high-aspect-ratio nanostructures and suspended nanowires on complex geometries	[191,197,198,199]
Scanning probe lithography (SPL)	Employs a sharp probe (such as in atomic force microscopy) to create patterns on a surface by mechanically removing material or inducing a chemical reaction	Rapid prototyping Extremely high resolution down to the atomic scale Capable of patterning various materials No need for complex masks or stencils Versatile in creating 2D and 3D structures Cost effective	Very low throughput Limited to small areas Requires precise control of the probe and surface Mainly a laboratory-based technique with limited commercial application	Development of nanoscale drug delivery systems Patterning of biocompatible surfaces Research in cellular interaction with nanostructures	[200,201,202]
Nanoimprint lithography	Involves pressing a nanostructured stamp into a polymer resist to create nanoscale patterns, then hardening the resist with thermal or UV curing, and transferring the patterns to a substrate through etching or lift-off	Cost-effective for large-scale production High resolution down to the nanoscale Simplicity Rapid prototyping Capable of producing 3D nanostructures Versatile with a wide range of materials	Challenging to achieve uniformity over large areas Requires high precision in alignment Limited by the mechanical properties of the stamp and resist Difficulties in pattern transfer for complex 3D structures Requires a resist that can withstand mechanical deformation	Patterning of surfaces for cell culture Fabrication of biosensors Creation of microfluidic devices Development of drug delivery systems Production of optical devices and components Fabrication of superhydrophobic and oleophobic surfaces	[191,203]
Electrospinning	Produces continuous nanofibers using a high-voltage electric field applied between a needle and a collector	High productivity Simplicity Low cost Reproducibility Functionality Diversity Potential in scaffolds with drug delivery systems and shape memory polymer materials Great fiber alignment based on collector design, promoting natural cell phenotype, migration, and proliferation Suitability of electrospun fibers as thin layers (epithelial, corneal stroma, and endothelial)	Dense electrospun fibers can hinder cell migration Not suitable for full-thickness corneal scaffolds due to limited thickness of electrospun membranes Low light transmission when synthetic polymer fibers are used	ECM biomimetic structures Tissue engineering Disease modeling Drug delivery systems	[179,181,185,204,205,206,207]
Two-photon polymerization (2PP lithography)	Direct laser writing technique that uses femtosecond laser pulses to create 3D structures within a photosensitive material	High-resolution method (capable of creating features smaller than the diffraction limit of light) Rapid prototyping and flexible (can create any 3D structure from computer models)	High cost Complex setup Sensitivity to laser fluctuations Limited material choices (only certain photoresists are suitable for 2PP) Need for more biocompatible photoinitiators and photoresists	Biomedical scaffolds Tissue engineering (mimicking native 3D environments such as Descemet’s membrane, luminal walls of blood vessels, etc.) Microfluidic lab-on-chip devices Drug delivery systems Creating master molds for subsequent replication with other techniques	[107,208,209,210,211,212,213,214,215]
Spincoating	Used to create thin film coatings by depositing a liquid on a spinning substrate, allowing centrifugal force to spread it uniformly	Simple, fast, and cost-effective High reproducibility and scalability Produces smooth and homogeneous films with controllable thickness (10 nm to several µm) Versatility: can produce monolayer- and multilayer-thin coatings, as well as freestanding (FS) films	Limited to planar surfaces Inefficient material usage (only 2–5% of material is used) Difficulties with large substrates Challenges in creating freestanding films	Biomedical applications (surface modification, drug delivery, wound dressings, tissue engineering scaffolds) Microelectronics	[216]

## 6. Emerging Technologies and Innovation

This section explores the potential of some of the latest advancements in tissue engineering for the development of corneal endothelium scaffolds, such as peptide and electroconductive hydrogels, 3D bioprinting, 4D bioprinting, drug delivery systems, and carbon nanotubes.

### 6.1. Peptide and Electroconductive Hydrogels

Hydrogels are a versatile biocompatible material that is now commonly employed in the biomedical field. They are a hydrophilic 3D network of crosslinked polymeric chains and can be made from natural or synthetic materials. Hydrogels are known for their ability to swell and adapt their volume in reaction to variations in environmental stimuli, as well as their drug delivery capacities [217].

Peptide hydrogels have the advantage of being able to spontaneously self-assemble, and the timing of this feature can be controlled with several physicochemical parameters [218]. In an effort to better understand polypeptides’ self-assembling mechanisms, Tang et al. (2019) developed a self-assembling pentapeptide triggered by pH and evaluated them as scaffolds through cell culture. The scaffolds showed promising results with cell growth. Although not studied in the context of corneal endothelium engineering specifically, their peptide hydrogel development demonstrates promise in the field of tissue engineering [219]. The 3D biofabrication of synthetic peptide hydrogel scaffolds has also been studied in many tissue engineering contexts, but has yet to be extensively explored in corneal endothelium tissue engineering [220].

Electroconductive hydrogels have also demonstrated great potential in tissue engineering, but they are less pertinent to the corneal endothelium, as they enhance electrical signaling in electroactive tissues [221]. Injectable hydrogels are another popular recent innovation known for facilitating drug delivery, which will be discussed further [222].

### 6.2. Carbon Nanotubes (CNTs)

Over the past decade, carbon nanotube (CNT) nanocomposite scaffolds have sparked significant interest within regenerative medicine by promoting neural, cardiac, muscle, and bone regeneration [223]. Whether single-walled or multiple-walled, they are known for their remarkable thermoelectrical conductivity and mechanical properties, with a tensile strength of around 0.85 GPa and a Young’s modulus of approximately 34.65 GPa [224]. These properties, along with their excellent biocompatibility, make them excellent candidates for fabricating robust scaffolds and drug delivery systems, with multiple-walled CNTs being the most suitable for carrying therapeutic agents [225].

Moreover, such scaffolds can also shape cell response and regeneration, with research demonstrating their ability to maintain the pluripotency of mouse iPSCs [226] to induce the growth and proliferation of human embryonic stem cells [227] and to promote better cell adhesion and spreading [228]. Combining collagen and CNTs has proven to be particularly effective in promoting cell growth [229]. Although the application of carbon nanotube scaffolds in corneal endothelium regeneration has yet to be explored, their unique features still make them a promising candidate in this area. However, an important limitation of CNTs is their cytotoxicity, which can be diminished with surface functionalization [223].

### 6.3. Three-Dimensional Bioprinting

Three-dimensional bioprinting is a young and evolving branch of regenerative medicine characterized by precise layer-by-layer deposition of a bioink, a printable material which can include cells, growth factors, and biomaterials to create three-dimensional tissue structures. The process is guided by a computerized image, and various printing methods are used, including inkjet, micro-extrusion, stereolithography (SLA), and laser-assisted bioprinting. In extrusion-based bioprinting, which is the most common printing method, bioink is loaded into a printing chamber and extruded through a nozzle to produce a continuous filament. If the printing process is successful, the cells in the synthetic tissue will multiply and behave similarly to cells in real tissues [230]. However, steep challenges regarding this 3D bioprinting method are replicating complex native biochemical environments and the potential destruction of cells in the bioink if the nozzle is too small or if the printing pressure is too high, as well as supplying sufficient nutrients and oxygen to the cells [231]. Nevertheless, bioprinting remains a promising approach in corneal regeneration and scaffold fabrication due to its high spatial resolution and ability to handle and deposit both living cells and biomaterials at the same time during the printing process. In the last four years, numerous experimental studies have attempted to 3D print the corneal stroma and the corneal epithelium [232,233,234,235,236,237,238,239,240]. However, there are very few studies available on the bioprinting of the corneal endothelium, as illustrated in Table 5.

Nevertheless, the field of 3D bioprinting for the corneal endothelium is rapidly advancing, with significant potential for clinical applications. Future research should focus on continued innovation in bioprinting methods, such as combining extrusion-based printing with other techniques like digital light processing (DLP) to improve the resolution and cell viability. Recently, this has been demonstrated by the development of a DLP-based multi-material bioprinting system [243], a hybrid 3D printing system integrating DLP and direct ink writing (DIW) [244], and a composable-gradient DLP-based bioprinting system with a microfluidic mixer [245]. These advancements have shown promising results, including superior cell viability, the creation of complex structures with functional gradients, detailed microarchitectures with enhanced mechanical properties, and precise control over bioink gradients. All of these systems have demonstrated several or all of these beneficial outcomes. Additionally, Gao et al. (2017) have introduced a multiphoton-excited 3D printing that generates extracellular matrix scaffolds with exceptional resolution and enhanced cell viability and mechanical properties. It was used to create a human-induced pluripotent stem cell-derived cardiac muscle patch (hCMP) in a murine model [246]. Furthermore, recent research has shown that hydrogel-based bioinks, such as alginate and methacrylated gelatin (GelMA), a decellularized ECM and silk-based bioinks are promising candidates in 3D bioprinting for tissue regeneration [220,247,248,249]. However, achieving the right viscosity for both cell support and printability, ensuring the mechanical integrity and shape fidelity of printed constructs, and maintaining high cell viability during and after the bioprinting process are remaining challenges. Crosslinking, blending polymers (e.g., blending alginate with high-molecular-weight polymers), and incorporating nanomaterials are effective strategies to balance these properties, and those avenues should continue to be explored [250,251].

### 6.4. Four-Dimensional Bioprinting

Four-dimensional bioprinting is another emerging field with applications in tissue engineering, drug delivery, and biomedical implants [252,253,254]. It combines the spatial precision of 3D printing with dynamic, stimuli-responsive materials in order to better address the need of emulating the complex behavior of living systems. These smart materials can change their shape, properties, or functions over time in response to environmental stimuli, such as temperature, pH, moisture, or light. Examples of those so-called smart materials are shape memory polymers [255,256,257,258], thermo-responsive polymers [259,260,261], and bio-inks [262], as well as electroactive polymers and magnetically responsive materials [263,264]. Intelligent stimuli-responsive hydrogels, particularly injectable ones, have also transformed tissue engineering repair and controlled drug delivery [264,265,266,267,268]. Despite the limitations of this technology, notably a complex design process, limited material options, and potential biocompatibility concerns [269], numerous studies have demonstrated its successful application or at least favorable results across various disciplines, including bone and soft tissue engineering, cartilage repair, cardiovascular applications, wound healing, and neural tissue engineering [270]. Four-dimensional bioprinting could also be beneficial for the fabrication of corneal endothelium scaffolds, as it would enable them to dynamically respond to changes in the corneal environment. However, research on 4D bioprinting in corneal applications is progressing, but very limited. Table 6 summarizes the available studies on developments in 4D bioprinting for corneal applications.

While no research specifically addresses 4D bioprinting for corneal endothelium scaffolds yet, the previous studies, which focus on scaffolds for the corneal epithelium and stroma, provide important insights into the methods and materials that can be employed to develop CEC scaffolds exhibiting the same favorable outcomes.

First, the cytocompatibility and proliferation of cells that the 4D printed chitosan-based stem scaffolds offer would promote CEC growth. Moreover, their temperature-sensitive properties would enable CECs to better adapt to the corneal environment, and their reduced corneal opacity and neovascularization would ensure the transparency of the implants.

Additionally, the high print resolution and harmonious integration of both macroscale and microscale structural features that are seen in the 4D printed anionic gelatin methacrylate hydrogels, as well as the ability of the 4D printed collagen-based hydrogel to change their shape in response to stimuli, would be other valuable additions to corneal endothelium scaffolds. That last characteristic would increase corneal endothelium scaffolds’ resilience to the changing ocular environment and would facilitate the scaffold’s implantation.

### 6.5. Scaffolds with Drug Delivery Systems 

Scaffolds with drug delivery systems are becoming more and more prominent in various medical fields, including ophthalmology. By enabling the controlled, localized, and sustained release of pharmaceutical agents, they maximize therapeutic benefits while reducing side effects and the need for frequent drug administration. For example, sustained release systems and smart carriers have been developed for glaucoma treatment, including poly(epsilon-caprolactone) devices and nanogel-based natural polymers that deliver Timolol Maleate [275,276]. Interpenetrating polymeric networks (IPNs) [277] and nanofibers [278,279,280,281,282] also show immense promise in overcoming obstacles in ophthalmic drug delivery, such as low bioavailability, rapid clearance, and the poor penetration of medication for a variety of ocular conditions.

The poor penetration of topical medication is a particularly important challenge for the corneal endothelium since it is the most posterior layer of the cornea. Addressing this issue is essential because this layer cannot regenerate naturally and consequently, untreated endothelial loss can lead to corneal blindness [283]. Carbon quantum dots (CQDs) are spherical, water-soluble, and non-cytotoxic nanoparticles that have emerged as a favorable solution to that limitation [284]. A study by De Hoon et al. (2023) demonstrated that positively charged CQDs are capable of transporting therapeutic agents from the corneal epithelium all the way to the endothelium, making them promising candidates for drug delivery. However, smaller CQDs with a length of 1–2 nm were more effective than bigger ones (7–8 nm) in that endeavor [283]. Other promising candidates are poly lactic/glycolic acid (PLGA) microspheres, which have been found by Koda et al. (2017) to be able to controllably release the rho-associated kinase inhibitor to treat corneal endothelial disease [285].

It is important to note that the benefits of incorporating drug delivery systems in corneal endothelium scaffolds are not restricted to improving therapeutic efficacy, but also include promoting cell adhesion, growth, and differentiation through the supply of growth factors or other supportive compounds. For instance, a study by Niu et al. (2014) showed that heparin-modified gelatin scaffolds for human corneal endothelial cells that absorb and gradually release fibroblast growth factors increase cell proliferation and viability [72]. Moreover, in a more recent study by Wang et al. (2023), an injectable double-network hydrogel that controllably releases hydrophilic and hydrophobic compounds effectively supported corneal regeneration [286].

Regarding techniques to create drug-delivering CEC scaffolds, 3D bioprinting, 4D bioprinting, and electrospinning are promising avenues. Three-dimensional and four-dimensional bioprinting have helped develop innovative and customizable drug delivery systems, with 3D bioprinting enabling personalized dosing [287] and 4D bioprinting allowing for the release of drugs only in response to appropriate physiological conditions [252,268,288,289,290,291,292,293,294]. Electrospinning is also largely used for drug delivery applications, whether in the cornea or other organs [204,295,296,297,298,299]. This is largely due to the scalability of this technique, as well as the high surface area-to-volume ratio, controlled release, encapsulation efficiency, and versatility in material choice that electrospun fibers can offer [300].

## 7. Challenges and Future Directions

Currently, studies on scaffolds for corneal endothelium tissue engineering are limited to preclinical trials. As there are many specific requirements to fulfill in the design of a functional scaffold, finding the right combination of materials to meet those criteria is a challenging task. There is also still a lack of understanding of corneal endothelial cells and the markers of a healthy and functional cell, which has to be guaranteed before introducing cultured CECs in a clinical setting [57]. Another common limitation to these preclinical studies is the regenerative capacity of rabbit corneal endothelial cells. Rabbit CECs and rabbit subjects are often employed to test new material advancements, but it is difficult to evaluate the true effect of the scaffold on cell growth and repair [72]. More studies on different test subjects such as cats or pigs are needed to better evaluate the viability of new materials, and there is still a long way to go before clinical trials can be safely performed.

Other areas would benefit from further investigation to fully harness the significant progress that has been made in biomaterials and fabrication techniques for corneal endothelium tissue engineering. First, although there have been significant advancements in creating hybrid biomaterials, finding more combinations that can achieve optimal transparency, biocompatibility, robustness, and permeability in scaffolds would be highly profitable. Furthermore, deepening our understanding of the various biological processes behind endothelial cell proliferation and topological morphology would lead to the fabrication of micro- and nanofeatures that more faithfully replicate DM and that better optimize cell adhesion, proliferation, and differentiation. Moreover, even though advanced manufacturing techniques like two-photon polymerization, electrospinning, 3D bioprinting, and 4D bioprinting are highly effective at creating complex scaffold structures, there is a gap between the utility of these methods and their practical application in corneal endothelium tissue engineering. More research needs to be conducted to fill that gap. The same applies for carbon nanotube (CNT) scaffolds and drug delivery systems. While CNT scaffolds have shown immense promise in promoting neural, cardiac, and bone regeneration and drug delivery systems in enhancing graft survival and therapeutic outcomes, there are limited studies that would facilitate their usage in corneal endothelium scaffolds. Other topics in need of deeper exploration are multicomponent membranes, which are membranes composed of various components or layers with distinctive properties, as well as combining manufacturing processes (e.g., melt electro-writing and melt electrospinning), as these have the potential to more accurately emulate living tissue and achieve complexity. Ultimately, further research in the previously mentioned areas, scaling up production and initiating early-phase human trials would all help the clinical translation of the in vitro and in vivo studies on corneal endothelium tissue engineering.

Other emerging areas of study not covered in this manuscript but which could open new future horizons include scaffold-free cell-based tissue engineering therapies [301], ultrasound-assisted tissue engineering [302], the use of bioresponsive materials and cellular reprogramming to allow for in situ tissue regeneration using the body’s own regenerative mechanisms [303], and the utilization of microfluidics to construct fibrous scaffolds [304]. Additionally, many scientists, such as Dalton et al. (2020), view the complete automation of hybrid manufacturing processes by robotization as likely to occur in the next two decades [153]. 

## 8. Conclusions

This review has provided a comprehensive overview of the advancements in polymer biomaterials for corneal endothelium tissue engineering, highlighting the essential properties required for scaffolds, including optical clarity, biocompatibility, mechanical stability, and permeability. We have discussed various natural, synthetic, and semi-synthetic polymers, along with innovative fabrication techniques such as electrospinning, 3D/4D bioprinting, and the incorporation of drug delivery systems, all of which are crucial in developing functional scaffolds for corneal endothelial cell (CEC) regeneration.

Despite the significant progress in this field, challenges remain, particularly in translating these technologies from the laboratory to clinical practice. However, the potential benefits of tissue engineering are immense, especially in addressing the global shortage of donor corneas. In developing countries, where access to donor tissues is particularly limited, the application of engineered corneal endothelia could revolutionize the treatment of corneal diseases, providing a more equitable solution for those suffering from vision impairment due to endothelial dysfunction.

## Figures and Tables

**Table 5:** Summary of the available experimental studies on 3D printing techniques of the corneal endothelium.

Corneal Layer	Corneal Endothelium	Corneal Endothelium
Year	2018	2024
Bioink Method	Extrusion-based 3D printing	Extrusion-based 3D printing
Bioink Substrate	Lyophilized amniotic membrane	Hydrazone crosslinked hyaluronic acid
Cell Source	Ribonuclease 5-overexpressing human corneal endothelial cells (HCECs) and control HCECs	Human pluripotent stem cell-derived corneal endothelial cells
Advantage	Improved cell confluency and functionality; enhanced corneal clarity and reduced edema	High viability and printability of cells; biocompatibility with various corneal tissues; good morphology and phenotype maintenance
Limitation	Potential for graft shrinkage; challenges with consistent cell attachment and survival	Some areas showed mesenchymal-like cell growth; more research needed to confirm adhesive properties in vivo
In vivo Study	No (ex vivo transplantation on rabbit corneas)	No (ex vivo transplantation on rat and porcine corneas)
Use with HCECs (Yes/No)	Yes	Yes
Reference	[241]	[242]

**Table 6:** Summary of recent advances in 4D bioprinting for corneal applications.

Year	Corneal Layer	Material/Technology	Applications	Study Type	Advantages	Challenges/Limitations	Reference
2024	Epithelium	4D printed chitosan-based thermosensitive hydrogel scaffold for rat limbal epithelium stem cells (LESCs)	Corneal alkali burns repair	In vitro	Excellent cytocompatibility, enhanced proliferation and differentiation of cells, temperature-sensitive properties for better adaptation to corneal environment, improved repair efficacy (lower corneal opacity, reduced neovascularization, and higher corneal epithelial wound healing rate)	Further research needed to confirm long-term effectiveness and integration in clinical settings	[271]
2024	Epithelium	4D printed chitosan-based scaffold with LSCs (limbal stem cells)	Treatment of corneal epithelium injury in diabetic rabbits	In vivo	Rapid wound healing, improved corneal nerve repair, significant decrease in inflammation, enhanced epithelialization	Potential issues with long-term integration and stability	[272]
2023	Thin membranous tissues which include the cornea, epidermis, and periosteum (corneal layer not specified)	4D printed anionic gelatin methacrylate (GelMA) hydrogels treated with cationic poly-l-lysine (PLL)	Generating cell-laden thin membranous tissues like the cornea, epidermis, and periosteum	In vitro	Charge-driven shrinking (which depends on the molecular weight of PLL) improves the resolution of printed structures to 65 µm, generation of macroscale and microscale tissues within the same construct	Cytotoxicity of higher molecular weight PLL, optimization needed for cell viability and shrinking capabilities	[273]
2019	Stroma	4D printed collagen-based hydrogel containing localized bio-actuators (contractile cells) that use a contraction-inhibiting peptide amphiphile	Creating a self-curving biomaterial through localized control of cell actuators	In vitro	Structural and mechanical properties are more similar to natural corneal tissue compared to flat 3D scaffolds; production of cornea-shaped, curved stromal tissue equivalents	Requires precise control of bio-actuator activity	[274]

## Data Availability

Not applicable.
